# Exploratory analysis of myocardial function after extracorporeal cardiopulmonary resuscitation vs conventional cardiopulmonary resuscitation

**DOI:** 10.1186/s13104-020-04982-x

**Published:** 2020-03-06

**Authors:** Joseph E. Tonna, Stephen H. McKellar, Craig H. Selzman, Stavros Drakos, Antigone G. Koliopoulou, Iosif Taleb, Gregory J. Stoddard, Josef Stehlik, Frederick G. P. Welt, James F. Fair, Kathleen Stoddard, Scott T. Youngquist

**Affiliations:** 1 Division of Emergency Medicine, University of Utah School of Medicine, 30 N 1900 E, Salt Lake City, UT 84132 USA; 2 Division of Cardiothoracic Surgery, University of Utah School of Medicine, 30 N 1900 E, 3C127, Salt Lake City, UT 84132 USA; 3 Division of Cardiology, University of Utah School of Medicine, Salt Lake City, USA; 4 Division of Epidemiology, University of Utah School of Medicine, Salt Lake City, USA; 5 Cardiovascular ICU, University of Utah, Salt Lake City, USA

**Keywords:** Myocardial recovery, Cardiac function, Extracorporeal cardiopulmonary resuscitation (ECPR), Ventricular unloading, Cardiac arrest

## Abstract

**Objective:**

Ventricular unloading is associated with myocardial recovery. We sought to evaluate the association of extracorporeal cardiopulmonary resuscitation (ECPR) on myocardial function after cardiac arrest. We conducted a retrospective exploratory analysis, comparing ejection fraction (EF) after adult cardiac arrest, between ECPR and conventional CPR.

**Results:**

Among 1119 cases of cardiac arrest, 116 had an echocardiogram post-return of spontaneous circulation (ROSC) and were included. Thirty-eight patients had ≥ 2 echocardiograms. ECPR patients had differences in age, hypertension and chronic heart failure. ECPR patients had a lower EF post-ROSC (24% vs 45%; *p *< 0.01) and were more likely to undergo percutaneous coronary intervention (25% vs 3%; p < 0.01). In multivariate analysis, only ECPR use (β-coeff: 10.4 [95% CI 3.68–17.13]; *p *< 0.01) independently predicted improved myocardial function. In this exploratory study, EF after cardiac arrest may be more likely to improve among ECPR patients than CCPR patients. Our methodology should be replicated to confirm or refute the validity of our findings.

## Introduction

Within the last several years, utilization of extracorporeal life support (ECLS) therapies has globally expanded [1, 2]. Studies have suggested a mortality benefit for use during cardiac arrest—known as extracorporeal cardiopulmonary resuscitation (ECPR)—compared to conventional cardiopulmonary resuscitation (CCPR) [3–6]. The potential effect on survival has dominated existing research [7–9], and a dearth of information exist that examine non-survival patient relevant outcomes, such as myocardial function.

Myocardial function is depressed after cardiac arrest, so therapies to improve it are relevant to patients in cardiac arrest [10, 11]. For acute cardiogenic shock, mechanical circulatory support has suggested a survival benefit, and is an area of active debate [5, 12, 13]. This is highly relevant to CCPR treated patients, as ventricular unloading does not universally occur after return of spontaneous circulation (ROSC). While the mechanisms of myocardial recovery after mechanical ventricular unloading are not fully understood, they may involve some combination of decreases in ventricular wall stress, increases in perfusion, and decreases in demand [14, 15]. In principle, ECPR plus coronary revascularization induces all of these changes in patients with acute ischaemic cardiac arrest. Accordingly, we assessed differences in myocardial function after cardiac arrest among patients managed with and without ECPR. We hypothesized that ECPR-treated patients would show worsened initial myocardial function after arrest, but greater improvements in myocardial function over time. We speculated that this could be due to mechanical myocardial unloading with temporary mechanical circulatory support after cardiac arrest.

## Main text

### Methods

#### Study design

We performed a retrospective cohort analysis of a prospectively-collected Utstein style database of patients who sustained out-of-hospital cardiac arrest (OHCA) within the Salt Lake City, UT region between 7/2008 and 12/2017. Data was extracted from the electronic medical records of patients directly into REDCap [16] by a trained quality assurance officer blinded to the goals of the analysis and supervised by the medical director (STY).

The ECPR program for OHCA began in June 2015, and targeted OHCA patients < 60 years of age, with witnessed arrest, initial shockable rhythm, bystander CPR and no significant known comorbidities, such as trauma, cancer, or organ failure, who had refractory cardiac arrest (no ROSC after ≥ 20 min of conventional advanced cardiac life support [17]. The decision is made based on initially reported criteria, whereas eventually confirmed patient characteristics may be different. ECPR data was similarly extracted by a trained research coordinator blinded to the goals of the analysis and supervised by the ECMO co-director (JET). The study was approved with a waiver of informed consent by the Institutional Review Board at the University of Utah.

#### Participants

Participants were identified for analysis if they had a cardiac arrest with ROSC, or return of spontaneous beat after ECPR, followed by an echocardiogram. Patients received an echocardiogram per routine clinical practice. Patient selection flowchart can be found in Additional file 1: Additional Digital Content. Patients were then separated by whether or not they were treated with ECPR as part of their resuscitation at the study hospital. The majority of ECPR patients had OHCA and were transported by EMS (n = 14) though a few patients sustained in-hospital/emergency department (ED) (n = 4) arrests. The CCPR cohort included patients transported by EMS to hospitals within the region (n = 9).

#### CCPR treatment

There was no explicit control of CCPR treatment, and patients were managed according to practice patterns at each institution, which typically included coronary angiography and revascularization in cases of ST-elevation on electrocardiograms after ROSC and targeted temperature management.

#### ECPR treatment

Components of treatment with ECPR in this cohort have been previously described [17], but include cannulation in the emergency department, coronary angiography with revascularization, targeted temperature management (36 °C) in the intensive care unit for 24 h, followed by controlled rewarming over 72 h.

#### Outcomes and covariates

The primary outcome was change in myocardial function, adjusted for time, measured on serial post-arrest echocardiograms. Change in myocardial function was defined as the magnitude and direction of change in percent ejection fraction (EF) from first to last echocardiogram among patients with ≥ 2 echocardiograms, post ROSC/return of spontaneous beat, analyzed as a continuous variable. For ECPR patients, to account for the relationship between ventricular “loading” and apparent function/EF, we limited analysis to studies obtained while on VA ECMO support. Echocardiographs were performed per routine clinical practice typically with < 2.5 L per minute of flow during the echocardiogram. To adjust for potential unequal spacing of studies, the outcome was normalized for time (hours) between studies. To assess clinically meaningful changes, we excluded repeat echocardiograms within 12 h. In order to account for the delayed period of observation among survivors (and thus myocardial recovery), we additionally analyzed the data after limiting to a period of 14 days post-cardiac arrest. Analysis was not limited to survivors, but rather to subjects with post-arrest echocardiograms. Recorded covariates included age, sex, past medical history (PMH), date of arrest, witnessed, initial rhythm, ECMO use, success of ECMO, echocardiographic EF with date/time, post-arrest coronary angiography, post-arrest percutaneous coronary intervention, and survival.

#### Statistical analysis

Descriptive statistics, including mean (standard deviation, SD) and median (interquartile range, IQR), were used to assess patient characteristics. Categorical characteristics were compared using Chi square test or Fisher’s exact test. Continuous characteristics were compared using independent samples t-test or Wilcoxon-Mann–Whitney test. We selected candidate predictors of myocardial recovery based on previously published studies examining ECPR survival. We additionally empirically selected other candidate predictors in our initial model. All were tested using Fisher’s exact test. We constructed univariate regression models for each potential confounder with the outcome. Variables with an association below 0.1 were considered as candidate covariates and included in the multivariate analysis if the association had biologic plausibility. For each potential confounder, we fit a separate regression model with the primary predictor and the potential confounder (covariate). Covariates that changed the *β*-coefficient of the primary predictor by ≥ 10%, were flagged as potential confounders [18] and added to the model. We then fit a multivariable linear model with significant confounders using either method. To test for multicollinearity, we assessed Pearson correlations (r) and the variance inflation factor (VIF), where a VIF > 2.5 indicated potential collinearity. No variables in the final model had a VIF ≥ 1.5. To test the durability of our findings, we performed sensitivity analyses limiting to echocardiographs obtained within 14 days of ROSC. Statistical analyses were conducted in STATA 15.1 (College Station, TX), and all reported *p* values are for two-sided comparisons.

### Results

#### Study population

One thousand one hundred and nineteen cases of adult cardiac arrest were identified since 7/2008. (Additional file 1: Figure S1) One hundred and sixteen cases survived to have an echocardiogram post-ROSC/return of spontaneous beat and were included in the initial analysis. Thirty-eight patients had ≥ 2 echocardiograms. Among all patients, the average age was 56 (SD 15.3) years old with 78% male. Eighty-six percent of the arrests were witnessed, and the initial arrest cardiac rhythm was ventricular fibrillation (VF) or ventricular tachycardia (VT) in 59%.

**Fig. 1:**
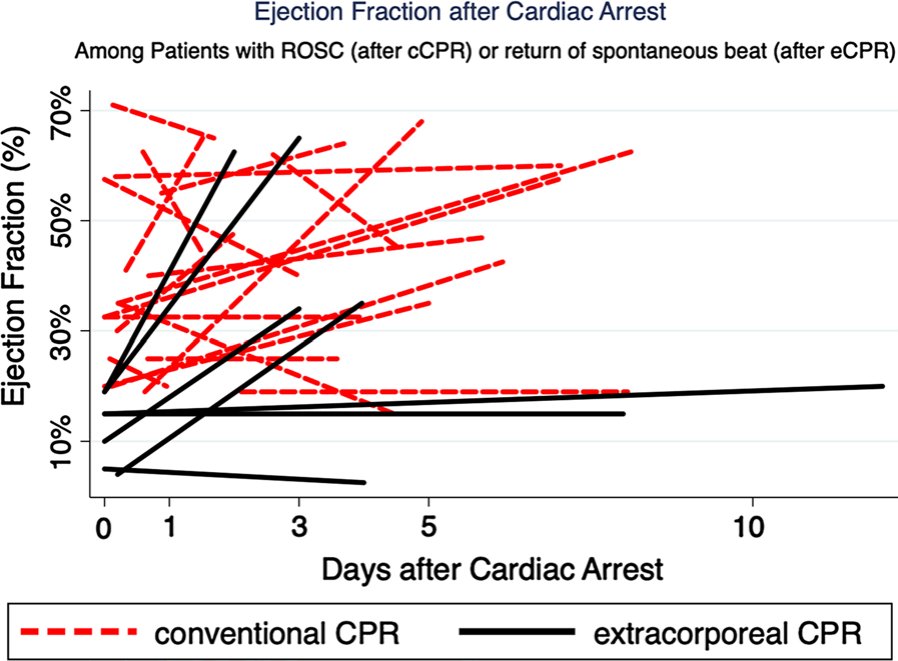
Thirty-eight (38) patients had ≥ 2 echocardiograms post-ROSC. Examining the ejection fraction (EF) over time (first to last) among the 26 patients who had ≥ 2 echocardiograms within 14 days post ROSC, divided by patients treated with ECPR (black line) and CCPR (red dashes). *CCPR* cardiopulmonary resuscitation, *ECPR* extracorporeal cardiopulmonary resuscitation, *ROSC* return of spontaneous circulation, *CPR* cardiopulmonary resuscitation

Patients placed on ECPR were less likely to a history of hypertension (8.3% vs 41.4%; p = 0.03). ECPR patients were non-significantly younger (49 vs 57 years; *p *= 0.08) and more likely to have a history of chronic heart failure (CHF) (33.3% vs 13.5%; *p *= 0.09). There were no significant differences in sex, witnessed arrest or first arrest rhythm. Patients placed on ECPR were also more likely to undergo post-arrest coronary catheterization (41.7% vs 8.7%; p < 0.01) and PCI (25% vs 2.9%; p = 0.01). (Additional file 1: Table S1) The initial post-ROSC EF was lower in ECPR patients (24% vs 45%; *p *< 0.01). The final EF was also worse among ECPR patients (33% vs 46%; *p *= 0.07). (Table 1, Fig. 1) The duration of time between sequential echocardiograms was non-significantly greater among CCPR patients (57.0 vs 5.1 days; *p *= 0.31), but when isolated to 14 days, this difference narrowed (3.9 vs 5.1 days; *p *= 0.31). Among ECPR patients, survivors had a worse initial EF (12.4% vs 40.4%; p < 0.01) compared to non-survivors.

**Table 1:** Echocardiographic characteristics

	ECPR	CCPR	*p* value
	n = 12	n = 104	
EF at first post-ROSC/return of spontaneous beat echocardiogram; % (± SE)	24% (5.2)	45% (1.8)	< 0.01
EF at last echocardiogram; % (± SE)^a^	33% (8.9)	46% (2.5)	0.07
EF at last echocardiogram; % (± SE)^b^	33% (8.9)	45% (3.9)	0.18
Duration of time between studies; days (± SE)^c^	5.1 (1.4)	57.0 (24.3)	0.31
Duration of time between studies; days (± SE)^b^	5.1 (1.4)	3.9 (0.5)	0.31

*CCPR* cardiopulmonary resuscitation, *ECPR* extracorporeal cardiopulmonary resuscitation, *EF* ejection Fraction, *ROSC* return of spontaneous circulation, *SE* standard error

^a^ In n = 38 patients with 2+ echocardiograms

^b^ In n = 26 patients with 2+ echocardiograms within 14 days

^c^ In n = 36 patients with dates for 2+ echocardiograms

In univariate analysis, ECPR use (*β*-coeff: 7.21 [95% CI − 1.13 to 13.28]; *p *= 0.02) and hypertension (*β*-coeff: − 4.61 [95% CI − 9.79 to 0.56]; *p *= 0.08) were associated with the outcome. (Table 2) In the multivariate linear regression analysis, a history of CHF was associated with worsened myocardial function (*β*-coeff: − 6.15 [95% CI − 12.11 to − 0.18]; *p *= 0.04), whereas ECPR use (*β*-coeff: 10.4 [95% CI 3.68 to 17.13]; *p *< 0.01) independently predicted improved myocardial function.

**Table 2:** Regression of predictors of time adjusted-myocardial function

Univariable	*β*-coef	95% CI	*p* value
ECPR	7.21	1.13 to 13.28	0.02
Age	− 0.04	− 0.23 to 0.16	0.71
Male sex	− 3.18	− 9.34 to 2.98	0.30
Shockable rhythm	0.47	− 5.05 to 5.99	0.86
Hypertension	− 4.61	− 9.79 to 0.56	0.08
Chronic heart failure	− 2.58	− 8.78 to 3.61	0.40
Duration between echocardiograms	− 0.01	− 0.03 to 0.02	0.62
Witnessed	5.04	− 4.36 to 14.44	0.28
Post-arrest coronary catheterization	1.4	− 3.86 to 6.76	0.54
Post-arrest PCI	− 0.58	− 7.56 to 6.40	0.87

Multivariable	*β*-coef	95% CI	*p* value
ECPR	10.4	3.68 to 17.13	< 0.01
Chronic heart failure	− 6.15	− 12.11 to − 0.18	0.04
Hypertension	− 3.22	− 8.08 to 1.63	0.19
Post-arrest PCI	− 4.32	− 10.79 to 2.13	0.18

*CI* confidence interval, *ECPR* extracorporeal cardiopulmonary resuscitation, *PCI* percutaneous coronary intervention

Although our primary outcome was adjusted for the uncontrolled time difference of echocardiographic assessments, we performed an additional sensitivity analysis after censoring the data at 14 days. In this model, CHF lost significance (*β*-coeff: − 5.63 [95% CI − 13.11 to 1.84]; *p *= 0.13), but ECPR use remained a significant predictor of myocardial function (*β*-coeff: 11.85 [95% CI 2.67 to 21.03; p = 0.01]).

### Discussion

We performed an exploratory analysis to examine change in myocardial function after cardiac arrest in patients managed with and without ECPR. We found that after controlling for relevant covariates in this preliminary analysis, myocardial function after cardiac arrest was more likely to improve among patients treated with ECPR than among patients treated with CCPR.

There are a number of plausible explanations for our findings. First, extrapolating from the bridge-to-recovery durable ventricular assist device population [14, 19], it is conceivable that improved myocardial function may result from a similar decompression of the left ventricle/cardiopulmonary tree via venous drainage into the ECLS circuit. In patients with mechanical circulatory support, one study demonstrated that the degree of left ventricular distension during ECMO is inversely associated with myocardial recovery [20], and another that the use of the Impella^®^ ventricular catheter simultaneously in patients on VA-ECMO was associated with lower mortality and inotropic support [21, 22]. This suggests a benefit of both direct and indirect unloading after cardiac arrest. Second, our ECPR protocol involves immediate coronary angiography and percutaneous coronary interventions (PCI), as warranted. As an appreciable proportion of cardiac arrests are due to acute coronary ischaemia [23], and PCI is itself independently associated with improved myocardial function and survival [24–28], we assessed this and found that PCI was not independently associated with myocardial function in our study. Thirdly, the use of the ECLS circuit enables perfusion independent of myocardial function. Thus, the need for inotropic and vasoactive medications may be less. This is important as it is known that inotropic medications increase myocardial oxygen demand during a period of limited supply [29, 30].

Notably, only a few ECPR patients underwent mechanical ventricular venting, as has been well-described in the acute cardiogenic shock population [22, 31–34], but the large majority achieved adequate decompression through ECMO flow modulation, inotropic use, and observed improved function after ECLS initiation and PCI. This is important, as if ECPR improves myocardial function through indirect cardiac unloading, then the natural extrapolation from the aforementioned literature is that protocoled ventricular venting may provide further improved outcomes [21, 34, 35].

Our study is important because it is the first of its kind, to our knowledge, to examine cardiac outcomes for ECPR. While a survival benefit remains unproven, we believe outcomes beyond survival are relevant in the determination of ECPR utility. We believe myocardial function to be of increasing importance after cardiac arrest, as surviving only to be limited by heart failure may impact quality-adjusted life years, economic productivity and patient-reported outcomes.

### Conclusions

Among a small population of patients (n = 38) who sustained cardiac arrest with return of circulation and serial echocardiograms, we found a preliminary signal that those treated with ECPR who achieved return of spontaneous beat had worse initial myocardial function compared to those achieving ROSC after CCPR. Among the ECPR patients, the improvement in short-term myocardial function, measured by ejection fraction, was significantly greater versus the CCPR population. These findings should be considered hypothesis generating and should be replicated in a larger population.

## Limitations

The primary limitations of this study were the selection bias of receipt of echocardiogram, which was dependent on ROSC and admission to the hospital (n = 379) (see Additional file 1: Additional Digital Content) and the small sample size. We were able to adjust for a number of important covariates. Other covariates to include in future validation studies include diastolic function and inotropic dosing.

## Supplementary information

**Additional file 1.** Supplemental digital content.

## Data Availability

To facilitate research reproducibility, replicability, accuracy and transparency, the datasets and the analytic code will be made available, following publication, in the Open Science Foundation (OSF) repository (10.17605/osf.io/msdf8, available at https://osf.io/MSDF8). Data were de-identified in accordance with Section 164.514 of the Health Insurance Portability and Accountability Act (HIPAA).

## Abbreviations

ECPR: Extracorporeal cardiopulmonary resuscitation
ECLS: Extracorporeal life support
CCPR: Conventional cardiopulmonary resuscitation
EMS: Emergency medical services
ED: Emergency department
OHCA: Out of hospital cardiac arrest
CHF: Chronic heart failure
PMH: Past medical history
VA ECMO: Veno-arterial extracorporeal membrane oxygenation
EF: Ejection fraction
ROSC: Return of spontaneous circulation
VF: Ventricular fibrillation
VT: Ventricular tachycardia
PCI: Percutaneous coronary intervention
PEA: Pulseless electrical activity
