# Supplementary material for: Exploratory analysis of myocardial function after extracorporeal cardiopulmonary resuscitation vs conventional cardiopulmonary resuscitation
Source: BMC Res Notes. 2020 Mar 6;13:137. doi: 10.1186/s13104-020-04982-x (PMC7060522; doi:10.1186/s13104-020-04982-x)
Supplement: Supplementary file 1 — Additional file 1. Supplemental digital content. [file 13104_2020_4982_MOESM1_ESM.docx]

**Exploratory analysis of myocardial function after extracorporeal cardiopulmonary resuscitation vs conventional cardiopulmonary resuscitation**

**ADDITIONAL DIGITAL CONTENT**

Joseph E. Tonna, MD

Stephen H. McKellar, MD, MS

Craig H. Selzman, MD

Stavros Drakos, MD, PhD

Antigone G. Koliopoulou, MD

Iosif Taleb, MD

Gregory J. Stoddard, MS

Josef Stehlik, MD

Frederick G. P. Welt, MD

James F. Fair III, MD

Kathleen Stoddard, RN

Scott T Youngquist, MD, MS

**Additional Methods**

*Patient selection:*

Among the 1,119 patients screened for inclusion, 11 were duplications, and 34 were <18 years of age at the time of arrest. Among the remaining 1,074 patients, 382 patients were pronounced dead in the field. Of those brought to the emergency department with on-going CPR, 313 had their resuscitation terminated in the ED. This left 379 patients for potential inclusion in the study who were eligible to receive an echocardiogram after ROSC.

*Criteria for echocardiograms and reporting:*

During the study period, echocardiograms were obtained as part of routine clinical care, guided by the clinical team, and without influence by study personnel. All echocardiograms were obtained by hospital certified echocardiographers and then interpreted for the medical record by hospital certified boarded cardiologists or anesthesiologists. These formal reads were then extracted.

Additional Results:

Comparing patients who received an echocardiogram, vs those that did not, there were no significant differences in age, end stage renal disease, cancer, cerebrovascular accident, hypertension, diabetes, chronic heart failure, respiratory failure, seizure, liver cirrhosis. Patietns were less likely to be female, have coronary artery disease, hyperlipidemia, or witnessed arrests. Correspondingly, they were less likely to have ventricular fibrillation and more likely to have asystole or non-shockable rhythms. Further notes of the significance of this potential bias can be found at the OSF at [https://osf.io/MSDF8].

**Additional Figure S1**: **Study enrollment flowchart**

**Additional Figure S1 Legend**

Among 1,119 patients who sustained cardiac arrest, 116 patients achieved return of spontaneous circulation (ROSC) and had a post-ROSC echocardiogram.

**Figure Abbreviations**

OHCA: Out of hospital cardiac arrest

ECPR: Extracorporeal cardiopulmonary resuscitation

CCPR: Cardiopulmonary resuscitation

ED: emergency department

**Additional Table S1: Demographics**

|  | **ECPR** | **CCPR** |  |
| --- | --- | --- | --- |
|  | **n=12** | **n=104** | ***p* value** |
| **Mean age ± SD (years)** [1 missing] | 49 (13.1) | 57.0 (15.4) | 0.08 |
| **Male, n (%)** | 8 (66.7%) | 82 (78.39 | 0.34 |
| **PMH, n (%)** |  |  |  |
| End Stage Renal Disease | 0 (0%) | 5 (4.8%) | 1 |
| Cancer | 0 (0%) | 7 (6.7%) | 1 |
| Stroke/Transient Ischemic Attack | 0 (0%) | 4 (3.9%) | 1 |
| Hypertension | 1 (8.3%) | 43 (41.4%) | 0.03 |
| Hyperlipidemia | 1 (8.3%) | 35 (33.7%) | 0.10 |
| Diabetes | 2 (16.7%) | 28 (26.9%) | 0.73 |
| Coronary Artery Disease | 4 (33.3%) | 28 (26.9%) | 0.74 |
| Chronic Heart Failure | 4 (33.3%) | 14(13.5%) | 0.09 |
| Chronic Obstructive Pulmonary Disease | 2 (16.7%) | 11 (10.6%) | 0.62 |
| Venous Thromboembolic Event | 1 (8.3%) | 4 (3.9%) | 0.43 |
| Seizure | 0 (0%) | 6 (5.8%) | 1 |
| Liver Cirrhosis | 0 (0%) | 0 (0%) | 1 |
| **Witnessed, n (%)** | 11 (91.7%) | 88 (85.4%) | 0.55 |
| **First Arrest Rhythm, n (%)** |  |  | 0.80 |
| VF/VT | 7 (58.3%) | 62 (59.6%) |  |
| Asystole | 2 (16.7%) | 11 (10.6%) |  |
| PEA/Other Non Shockable | 3 (25%) | 31 (29.8%) |  |
| **Coronary Cath Performed, n (%)** | **5 (41.7%)** | **9 (8.7%)** | <0.01 |
| **PCI, n (%)** | 3 (25%) | 3 (2.9%) | 0.01 |

CCPR: Cardiopulmonary resuscitation

ECPR: Extracorporeal cardiopulmonary resuscitation

SD: Standard deviation

PMH: Past medical history

VF/VT: Ventricular fibrillation/ventricular tachycardia

PEA: Pulseless electrical activity

Cath: Catheterization

PCI: Percutaneous coronary intervention
